# Collaborative pharmacy research across integrated health systems: A purpose and promise for opportunities to study the complete medication-use process

**DOI:** 10.1093/ajhp/zxae266

**Published:** 2024-10-14

**Authors:** Anthony W Olson, Michael J Miller, Pamala A Pawloski, Stephen C Waring, Jennifer L Kuntz, Xiaojuan Li, Jenna Wong, Eric A Wright

**Affiliations:** Research Division, Essentia Institute of Rural Health, Duluth, MN, and Department of Pharmacy Practice and Pharmaceutical Sciences, College of Pharmacy, University of Minnesota, Duluth, MN, USA; Mid-Atlantic Permanente Research Institute, Rockville, MD, USA; HealthPartners Institute, Bloomington, MN, and Department of Experimental and Clinical Pharmacology, College of Pharmacy, University of Minnesota, Minneapolis, MN, USA; Research Division, Essentia Institute of Rural Health, Duluth, MN, and Department of Pharmacy Practice and Pharmaceutical Sciences, College of Pharmacy, University of Minnesota, Duluth, MN, USA; Kaiser Permanente Center for Health Research, Portland, OR, USA; Department of Population Medicine, Harvard Medical School, Boston, MA, and Harvard Pilgrim Health Care Institute, Boston, MA, USA; Department of Population Medicine, Harvard Medical School, Boston, MA, and Harvard Pilgrim Health Care Institute, Boston, MA, USA; Center for Pharmacy Innovation & Outcomes, Geisinger, Scranton, PA, and Department of Bioethics and Decision Sciences and Department of Pharmacy, College of Health Sciences, Scranton, PA, USA

**Keywords:** integrated health systems, managed care organizations, medication-use process, outcomes research, research methods, research networks

Prescription medications are commonly used to help diagnose, prevent, treat, and manage disease. Advancements in drug design, development, and clinical monitoring continue to contribute to decreased morbidity and mortality as well as improved quality of life for patients across the spectrum of diseases.^1-4^ Nearly two-thirds of US adults took at least one prescription medication in 2022, and 25% were taking 4 or more.^5^ Further, US adults obtained 6.4 billion prescriptions in 2022, accounting for ~10% (more than $4 billion) of healthcare expenditures.^6,7^ Importantly, these costs do not reflect the unintended harmful events associated with normal use of medications (ie, adverse drug events, or ADEs).^8^ ADEs are responsible for roughly 5 million emergency department visits annually and by some estimates are the third most common cause of death in the US behind heart disease and cancer.^9-12^ Approximately half of ADEs are believed to be preventable.^13^

The importance and value of prescription medications are tied to their ubiquity, cost, and potential benefits and harms. These key domains contain critical unresolved research questions around the medication-use process (MUP) relevant to policy and practice.^14^Figure 1 consolidates prominent MUP models, consisting of 4 key domains of (de)prescribing/transcribing, preparing/dispensing, taking/administering, and monitoring/follow-up.^15-18^ Selection/procurement, safe storage, and disposal/waste are also important elements within the process.^16,17,19,20^ Each of these domains and elements generates important research questions.

**Figure 1.**
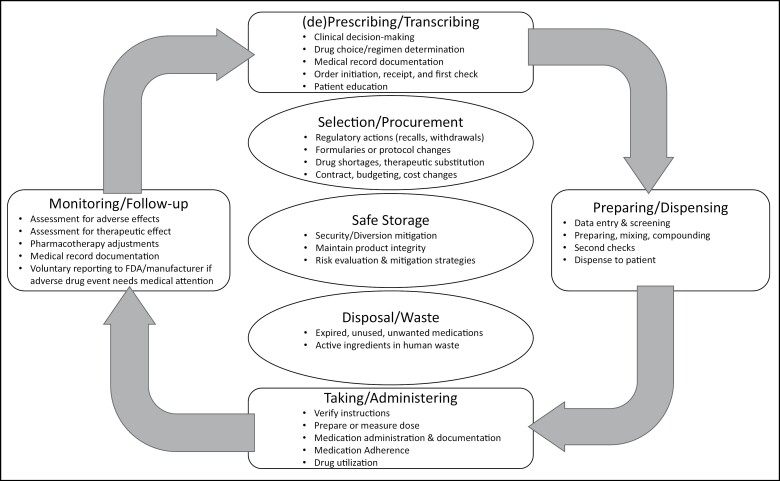
Merged medication-use process (adapted from multiple sources).^15-20^ Rectangular shapes correspond to the domains most commonly represented across all medication-use process frameworks. Oval shapes are elements with ties to several of the cycle domains and inherently adjacent to their activities. FDA indicates Food and Drug Administration.

Numerous translational studies have focused on the aforementioned MUP domains and elements to enhance practice, patient-clinician communication, and the medication-use experience.^21-26^ Several important MUP-based studies were conducted by practice-based research networks led by academic pharmacy^27-29^ and clinically integrated practice networks of independent/small-chain pharmacies.^30^ These important contributions advancing MUP research have been challenged by several limitations. All research and practice networks face challenges of funding network activities, training staff to conduct collaborations, and protecting time for clinicians and researchers to engage in research activities. Academic-based and independent/small-chain pharmacy networks have additional barriers to overcome such as developing processes for primary data collection (eg, identification, recruitment, and enrollment of study participants from numerous geographically dispersed organizations); ensuring the accuracy, completeness, and standardization of data and obtaining funding to acquire secondary databases (eg, administrative or claims); and addressing the unique requirements, goals, and needs of member sites (eg, data storage, access, ownership, and relevance to mission).^31-36^ While technology-driven solutions can help mitigate these challenges,^37^ sizable gaps remain in understanding the complex evidence enabling timely and informed ways to address persisting real-world MUP problems.^1,15,22,38-42^

Since the 1990s, the US government has supported the formation and development of several research consortia embedded in collaborative and integrated community-based health systems. These collaborations have successfully addressed key gaps in research areas,^43^ including in cancer,^44,45^ cardiovascular disease,^35^ integrated care models,^46^ mental health,^47,48^ and postmarketing surveillance of drugs, devices, and vaccines.^49-54^ Many of these initiatives were not led by pharmacists and pharmacy-based researchers, despite their formal training, expertise, and experience in the MUP. Research networks among integrated health systems can complement and collaborate with existing academic and practice networks to address challenges, barriers, and gaps in MUP research. Additionally, pharmacists and pharmacy-based researchers of varying backgrounds embedded in health systems are increasingly tasked by health-system leadership with operationalizing medication optimization methods. Thus, pharmacists and pharmacy-based researchers are well positioned to lead these collaborative efforts and drive needed change for medication therapy optimization. This commentary aims to (1) describe the purpose and promise of health system–based research networks with a focus on the MUP; (2) outline the pathway for the development of and participation in one such consortium; and (3) identify foreseeable challenges critical for this initiative to overcome.

## Purpose and promise.

Health-system research networks, often integrated with insurance providers and payors, have inherent advantages for conducting rigorous, multicenter, community-based MUP research of interest to extramural funders and policymakers.^43,44^ First, these networks generate data (1) collected through dynamic electronic health records (EHRs) and stored in research-ready data warehouses; (2) from large and diverse populations beyond those available at a single health system; (3) from stable populations (ie, patients are more likely to enroll and remain in studies conducted at their home health systems or integrated health plans); (4) across transitions between outpatient and inpatient care; and (5) representing real-world medication use.^45,55^

Second, many health systems have embedded research teams, centers, and institutes that provide critical support, inspiration, and outlets for research collaborations.^43,56^ As such, they are dedicated to rapidly identifying research questions and designs of practical and timely significance, quickly responding to funding opportunities, and seamlessly translating findings and effective interventions into practice. The nature of embedded research teams facilitates early and frequent collaboration among practitioners, administrators, and scientists. These teams use real-world data to identify care gaps and inform interventions to meet the needs of patients within the existing workflows of health systems, providers, and payors. This structure also enables collaborations across integrated health systems. Thus, a multi-stakeholder alignment of integrated health-system research networks benefits both individual health systems and the overall consortium, given that many have similar goals and philosophies, challenges, resource environments, infrastructure, and other characteristics.^43,44,57^

Third, integrated research networks use common data models (CDMs), which standardize the organization, coding structures, and variable definitions of datasets to support distributed processing of data.^58-61^ CDMs enable consistent measurement and analysis of patient (eg, race/ethnicity and health-related social needs), intervention, and outcome (eg, care quality, quality of life, cost-effectiveness, and subpopulations) variables over long time horizons because patients are more likely to remain with the same health system over time.^43^ These measurement qualities are useful to study topic areas such as the ever-changing and perplexing intersection of medication selection and reimbursement policies, finding and acquiring medications, and making medications available to the individuals or groups who need them. CDMs also enable EHR-sourced data to be efficiently used across different sites for short-term pilot and cross-sectional studies as well as longer-term and longitudinal cohort studies^62-65^ and interventional pragmatic studies.^66-69^ Similarly, use of a CDM across integrated networks expands generalizability through more complete and consistent capture of the numerous data variables related to medications, patients, providers, and health systems throughout the MUP that may influence therapeutic plans and outcomes.

The scope of most MUP studies is limited by the amount and type of data available to researchers. The results are understandings that may be partial at best and inaccurate at worst, and such studies are also limited by an inability to study rare medical conditions or ADEs. For example, studies on the prescribing or ordering process often do not account for any aspect of administration, medication adherence, monitoring, or outcomes. Claims-based studies focus on filled prescriptions and do not consider prescribing and primary or secondary medication adherence.^70-72^ Moreover, claims-based studies are often historical and do not allow for communication with providers or current patients to gain in-depth insight into the medication-use experience. Claims-only studies often lack the richness of medical and laboratory record data captured within EHRs, such as indication, therapeutic effectiveness, safety, and convenience/adherence documented from medication therapy management services provided by pharmacists. Making decisions impacting medication policy based on incomplete information may lead to misinformed decisions, suboptimal outcomes, and/or unintended consequences.

The overarching value of research within an integrated health-system network is the ability to study the entire medication-use continuum in a scalable and generalizable way through access to combined historical, real-time, and prospective patient data when they are most relevant and useful. In addition, a properly structured CDM enables researchers, practitioners, and patients to answer multiple medication-related research questions without recreating infrastructure for each individual research project or public health surveillance initiative (eg, surveillance of ADEs by the Food and Drug Administration Sentinel Initiative).^36,53,73^ A similar approach can be taken to conduct research in all domains of the MUP, such as when assessing the following:

- **(De)prescribing/transcribing:** collaborative practice agreements, biomedical informatics in clinical decision-making, comprehensive medication management
- **Preparing/dispensing:** medication synchronization, pharmacy workflows (eg, robotics, point-of-care vending for inpatients), mail-order pharmacy
- **Taking/administering:** socio-behavioral aspects of medication (eg, medication adherence, medication experience), drug infusion services, site of care, pharmacoepidemiology (eg, drug utilization)
- **Monitoring/follow-up:** pharmacovigilance, medication therapy management, pharmacoepidemiology (eg, comparative safety and effectiveness)
- **Selection/procurement:** pharmacoeconomics and policy (eg, specialty drugs, drug formulary management, contracting/pricing, drug shortages/supply chain issues)
- **Safe storage:** risk evaluation, drug security/diversion mitigation
- **Disposal/waste:** public health, drug disposal programs, environmental impact

## Connecting pharmacy research through the Health Care Systems Research Network.

**Figure 2.**
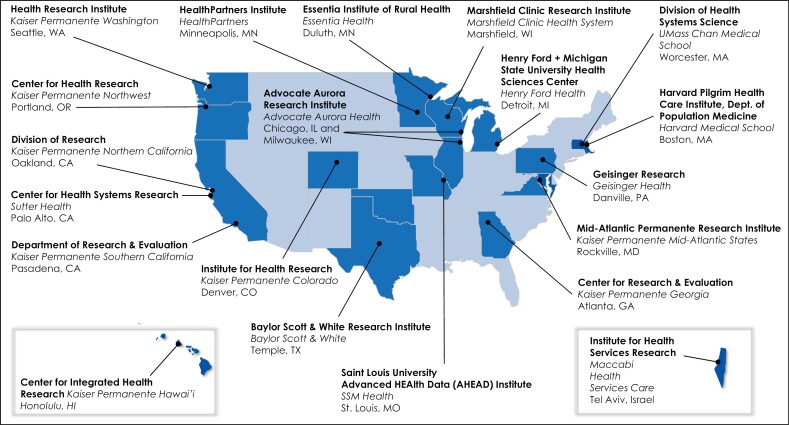
Locations and names of Health Care Systems Research Network (HCSRN) health-system members.^74^ Used with permission of HCSRN.

One of the largest networks of research centers embedded within health systems is the Health Care Systems Research Network (HCSRN), founded in 1994 and formerly known as the HMO Research Network.^36,43,57^ The organization is composed of 20 unique nonprofit integrated health systems providing healthcare for over 20 million US patients and connects data and researchers across institutions. The HCSRN generates public domain research, representing over 400 million person-years and in some cases extending back to 1958 (Figure 2).^74^ The HCSRN developed a first-of-its-kind CDM, the Virtual Data Warehouse (VDW), complemented by streamlined administrative processes that include institutional review board ceding, standardized data use agreements and research oversight, and shared resources and capabilities to efficiently collaborate on research to improve individual and population health for all. The network is made up of over 2,000 multidisciplinary research scientists and research staff at its member organizations, with scientific interest groups in aging, cancer, mental health, patient engagement, research administration, and, most recently, pharmacy.

The pharmacy scientific interest group (PhIG) was founded in 2022 and formed a workgroup to better organize the pharmacy-related data captured in HCSRN’s VDW. The purpose of HCSRN’s PhIG is to bring together individuals with expertise in pharmacy-related care, health outcomes, epidemiology, health services, implementation science, data management (eg, extraction, transformation, and loading) and analysis, and other disciplines who have an interest in sustainably exploring, evaluating, and improving medication-use data and processes. Consistent with the mission of the HCSRN, the PhIG aims to be as inclusive as is meaningfully possible. Participants from non-HCSRN member institutions are invited to join, and eligibility is not limited to pharmacists, pharmacy technicians, or solely pharmacy-specific researchers (inquiries may be sent to PhIG@hcsrn.org). PhIG’s immediate strategic goals are to (1) conduct ongoing VDW data enhancement and development of capabilities to capitalize on group member strengths and interests in pharmacy-related research and then (2) leverage the resulting functionality to disseminate research aligned with the group’s mission. Once these goals are achieved, PhIG’s strategic goal will shift to supporting established research subgroups pursuing and obtaining funding from federal and other extramural sources (eg, foundations) to sustain larger-scale projects.

## Navigating key challenges.

Realizing the promise of collaborative pharmacy research across integrated health systems requires awareness of and mitigation of at least 3 key challenges. First, the collaboration needs to benefit all intra- and interorganizational stakeholders and yield research results that are important and relevant to health systems, health plans, provider groups, research cohorts, patients, families, and communities. Views about how to best use and share data and stakeholder resources (eg, protection of clinician time for research and investments in infrastructure, technology, and staff) may not uniformly overlap. Substantial time and effort must be devoted to collectively aligning interests and incentives to preserve a culture and environment that promotes collaboration and pursuit of sustainable research funding while also producing meaningful knowledge relevant to stakeholders.

Second, the fast-changing nature of clinical, operational, and research environments requires continual vigilance, communication, and adaptation among participating members. The impact of changes to practice guidelines, payor policies, leadership structures, rules and laws, market dynamics, and other variables has complex and far-reaching implications that require ongoing and effective information sharing and decision synchronization. This also represents an opportunity for a functional research network.

Third, recruitment, coordination, and sustained engagement of researchers with aligned interests and goals in MUP research is another challenge. An effective research network must balance the niche of constituent interests with the most important and relevant MUP knowledge and implementation gaps prioritized by patients, providers, policymakers, and other key stakeholders.

## Conclusion and call to action.

Collaborations with embedded researchers within integrated community-based health systems, such as HCSRN, offer established research structures and real-world data that can address important research gaps. HCSRN’s PhIG provides a ready and available avenue for pharmacy-related researchers to participate in pharmacy research across integrated health systems. We invite interested parties to contact us at PhIG@hcsrn.org.
